# Supplementary material for: Multi-omics network reconstruction with collaborative graphical lasso
Source: Bioinformatics. 2026 Jul 13;42(7):btag477. doi: 10.1093/bioinformatics/btag477 (PMC13378458; doi:10.1093/bioinformatics/btag477)
Supplement: btag477_Supplementary_Data [file btag477_supplementary_data.pdf]

Bioinformatics, 2022, 1–14
doi: DOI HERE
Advance Access Publication Date: Day Month Year
Paper

Supplementary data to "Multi-omics network reconstruction with collaborative graphical lasso"

Alessio Albanese,[1,2] **Wouter Kohlen**[2] **and Pariya Behrouzi**[1,*]

[1]Mathematical and Statistical Methods group - Biometris, Wageningen University and Research, PO Box 16, 6700AA, Wageningen The Netherlands and [2]Cell and Developmental Biology, Wageningen University and Research, PO Box 633, 6700 AP, Wageningen, The Netherlands

*Corresponding author. pariya.behrouzi@wur.nl

FOR PUBLISHER ONLY Received on Date Month Year; revised on Date Month Year; accepted on Date Month Year

Abstract

Here we report the Supplementary Data to the article "Multi-omics network reconstruction with *collaborative graphical lasso*".

1. Algorithms

1.1. Update rule of the coordinate descent algorithms of *glasso*

The coordinate descent procedure described in Friedman et al. [2007] estimates $\widehat{\boldsymbol{\beta}}_{\boldsymbol{i}}$ from Equation 1 of the main text, for any i, by iteratively updating the j-th coordinate of the vector, $(\widehat{\boldsymbol{\beta}}_{\boldsymbol{i}})_j$, which is the main proxy for the conditional dependence between node i and node j of the network. The Algorithm begins with

Algorithm 1 *glasso* coordinate descent rule

> **for** $j = 1, 2, \ldots, p, 1, 2, \ldots, p, \ldots$, until convergence criterion is met **do**
> $r \leftarrow \boldsymbol{S}_{ji} - \sum_{k \neq j} \left(\boldsymbol{W}_{\setminus i \setminus i}\right)_{jk} \left(\widehat{\boldsymbol{\beta}}_{\boldsymbol{i}}\right)_k$
> **if** $r < -\lambda$ **then**
> $\left(\widehat{\boldsymbol{\beta}}_{\boldsymbol{i}}\right)_j \leftarrow \frac{r+\lambda}{\boldsymbol{W}_{jj}}$
> **else if** $r > \lambda$ **then**
> $\left(\widehat{\boldsymbol{\beta}}_{\boldsymbol{i}}\right)_j \leftarrow \frac{r-\lambda}{\boldsymbol{W}_{jj}}$
> **else**
> $\left(\widehat{\boldsymbol{\beta}}_{\boldsymbol{i}}\right)_j \leftarrow 0$
> **end if**
> **end for**

computing an initial value r. The value assigned to r is computed using two terms. The first one is the entry $\boldsymbol{S}_{ji}$ of the empirical variance-covariance matrix, the common variability of node i and node j. This common variability is adjusted for the role that all the other nodes k of the network exert on node i and on node j by subtracting a dot product to $\boldsymbol{S}_{ji}$. This reflects the principles of conditional dependence described above. After r is computed, its value can either fall outside or inside of the penalty interval $(-\lambda, \lambda)$. In the first case, the algorithm assigns to $(\widehat{\boldsymbol{\beta}}_{\boldsymbol{i}})_j$ the value of r shrunk towards the interval. In the other, $(\widehat{\boldsymbol{\beta}}_{\boldsymbol{i}})_j$ is fixed to 0. The updates are run multiple times for $j = 1, 2, \ldots, p, 1, 2, \ldots, p, \ldots$ until convergence of the vector $\widehat{\boldsymbol{\beta}}_{\boldsymbol{i}}$.

1.2. Update rule of the coordinate descent algorithms of *coglasso*

The chosen algorithm to solve the objective function of *collaborative graphical lasso* shown in Equation 2 of the main text is also a coordinate descent procedure. Here follows the update rule of the procedure, used by *coglasso* to estimate the j-th element of $\boldsymbol{\beta}_{\boldsymbol{i}}$, which represents a proxy for the connection between the i-th and the j-th variable. For the details about the derivation, we refer the reader to the Appendix A of these Supplementary Data. To keep the update rule more general, let j belong to a generic indexes set A, which could, for instance, represent variables from the omics layer $\boldsymbol{X}$.

Algorithm 2 *coglasso* coordinate descent rule

> **for** $j = 1, 2, \ldots, p, 1, 2, \ldots, p, \ldots$, until convergence criterion is met **do**
> $r \leftarrow \alpha \boldsymbol{S}_{ji} - \sum_{k \in A, k \neq j} \left(\boldsymbol{W}_{\setminus i \setminus i}\right)_{jk} \left(\widehat{\boldsymbol{\beta}}_{\boldsymbol{i}}\right)_k - \alpha(1-c) \sum_{k \notin A} \left(\boldsymbol{W}_{\setminus i \setminus i}\right)_{jk} \left(\widehat{\boldsymbol{\beta}}_i\right)_k$
> **if** $r < -\alpha \boldsymbol{\Lambda}_{ji}$ **then**
> $\left(\widehat{\boldsymbol{\beta}}_i\right)_j \leftarrow \frac{r+\alpha\boldsymbol{\Lambda}_{ji}}{\boldsymbol{W}_{jj}}$
> **else if** $r > \alpha \boldsymbol{\Lambda}_{ji}$ **then**
> $\left(\widehat{\boldsymbol{\beta}}_i\right)_j \leftarrow \frac{r-\alpha\boldsymbol{\Lambda}_{ji}}{\boldsymbol{W}_{jj}}$
> **else**
> $\left(\widehat{\boldsymbol{\beta}}_{\boldsymbol{i}}\right)_j \leftarrow 0$
> **end if**
> **end for**

© The Author 2022. Published by Oxford University Press. All rights reserved. For permissions, please e-mail: journals.permissions@oup.com

Here, $\alpha = \frac{1}{1+c}$. There are remarkable differences with *glasso*'s update rule seen in Algorithm 1 to update $(\widehat{\beta}_{\boldsymbol{i}})_j$. First, here entry $\boldsymbol{S}_{ji}$ is weighted by α. Second, when adjusting for the influence that nodes k exert on the connection between node i and node j, *coglasso* differentiates between two groups of nodes: nodes that belong to the same set of j ($k \in A$), and nodes that do not ($k \notin A$). When a node k belongs to the same set as j, the adjustment for the role of those nodes always has the same weight and direction, like for *glasso*. When instead it doesn't, the weight and direction of the adjustment depends on the c parameter. When $c = 0$ there is no difference between *coglasso* and *glasso* in computing r, and so in adjusting $\boldsymbol{S}_{ji}$ and the two adjustments for $k \in A$ and $k \notin A$ merge into a single update rule. For $c > 0$, collaboration activates, and the role of the variables $k \notin A$ on the connection between node i and j is adjusted. For $0 < c < 1$, the direction stays the same as the other group of nodes, but the weight of the adjustments slowly decreases. For $c = 1$, the weight of these nodes in adjusting the dependence between node i and node j is 0. For values of c higher than 1, the direction of the adjustment for the nodes that do not belong to the same omics layer as j inverts the direction, increasing its weight as the value of c increases. A final difference is that in *coglasso* the penalty interval is now defined as $(-\alpha\boldsymbol{\Lambda}_{ji}, \alpha\boldsymbol{\Lambda}_{ji})$. Then, similarly to *glasso*, if r falls out of it, we assign to $(\widehat{\beta}_{\boldsymbol{i}})_j$ a value closer than r to the interval, while if r falls inside it we assign 0 to it.

1.2.1. Collaboration and the c hyperparameter

As Algorithm 2 shows, collaboration affects the sparsity *within* and *between* the omics layers in the estimated network in a less direct way than λ_w and λ_b, acting more generally over the estimation of every edge of the network. When estimating the connection from a node i to a node j (i—j), the c value affects the process in two ways:

1. Directly influencing the sparsity through scaling Λ_{ij} by α ($\alpha = 1/(1+c)$). This happens uniformly across the whole network, regardless of i—j being a between or a within connection, with the only changing factor here being the Λ_{ij} value used (λ_w if i—j is a *within* connection, or λ_b if it is a *between* connection).
2. Altering the effect on the edge i—j of other variables k that are part of a layer different than the layer of variable j. With the traditional *glasso* approach, such variables are taken into account in the same way and measure of those that are part of the same layer as that of variable j. This can be obtained also for *coglasso* when $c = 0$, imposing the variables of layers different than j's to have the same role in the estimation of i—j as those that are part of the same layer. This changes the moment in which $c > 0$, and the role of such variable is taken into account differently, activating collaboration.

This complex role of of collaboration and of the c value justifies why, for example, in Supplementary Figure S3 some of the best networks may still benefit from collaboration being active, even when the simulated networks have between layer density of 0%. For instance, when i—j is a *within* connection of one of the two layers, and when the other layer is significantly larger in size, it is important to alter the effect of nodes that are part of the larger layer over such connection. Hence, even though c can affect the sparsity of the network, it does so in a less direct way than the values of λ_w and λ_b, and not necessarily with the same intensity between and within network. An example of the role of c over the structure of a multi-omics network built on real data is provided is Supplementary Figure S10. As for the the c values worth exploring, it is useful to observe how different values of c affect the $k \notin A$ term (effect of variables k that are not part of the same set as j over the i—j collection) in the rule for the computing of r in Algorithm 2. There, the weight applied to the term is $\alpha(1-c) = \frac{1-c}{1+c}$. Supplementary Figure S1 shows the value of the weight based on c.

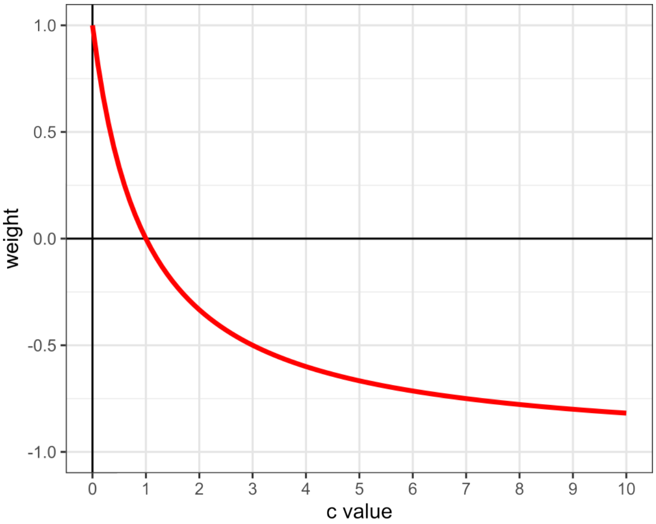

Supplementary Figure S1: Value of $\alpha(1-c) = \frac{1-c}{1+c}$ for increasing c value.

The function equals 1 for $c = 0$ (inactive collaboration), 0 for $c = 1$, and tends to -1 for $c \to +\infty$, even though already for $c = 100$ the value of the function is close to -0.98. Moreover, for reciprocal values of c, this function yields opposite weights. As a consequence, an fairly complete array of c values we would generally recommend exploring would generally include $c = 0$, $c = 1$, $c = 100$, and other reciprocal values symmetrically chosen around 1, starting usually from $c = 0.01$ (reciprocal of 100) for a light activation of collaboration.

If this clarifies a meaningful range of explorable c values, we recommend to select the optimal one simultaneously with the λ_w and λ_b values with the model selection procedure *XStARS* described in the next Section.

1.3. eXtended Stability Approach to Regularization Selection (*XStARS*)

Here we report our generalization of the *StARS* model selection procedure developed in Liu et al. [2010], *XStARS*. *XStARS* adapts *StARS* to select hyperparameters in the three-dimensional hyperparameter space of *coglasso*.

Algorithm 3 describes the outline of *XStARS*, with $\boldsymbol{c}$, $\boldsymbol{\lambda_b}$, and $\boldsymbol{\lambda_w}$ being the arrays of explored values for the hyperparameters. Here, the function $StARS(\boldsymbol{h})$ represents the same one-dimensional algorithm developed for *glasso*. It returns the hyperparameter value h in an array $\boldsymbol{h}$ yielding the most stable, yet sparse *coglasso* network, after fixing the other two hyperparameters to a given value. In other words *XStASRS* alternately estimates an optimal value for λ_w, λ_b, or c, keeping the other two fixed

to the last optimal value obtained. This alternation continues until one of two conditions is met. The first one is if the algorithm converges to a stable triplet of hyperparameter values $(\widehat{\lambda}_w, \widehat{\lambda}_b, \widehat{c})$ yielding the optimal stable, yet sparse network. In practice, for this happens when twice in a row the selection of two hyperparameters yields the same values it selected in the previous iteration of the algorithm. Alternatively, the alternation continues until the algorithm has cycled over the three hyperparameters for a predetermined maximum number of iterations, yielding the last triplet it selected. We choose as a default 10 iterations, an empirically chosen value that offers a good trade-off between stability and computational cost.

Algorithm 3 *XStARS*

```
MAX_ITER ← 10 (as a default value)
converged ← 0
λ̂_w ← −1
λ̂_b ← min(λ_b)
ĉ ← Max(c)
select_λ_w ← TRUE
select_λ_b ← FALSE
iter ← 0
while converged < 2 AND iter < MAX_ITER do
    if select_λ_w then
        λ_w^temp ← StARS(λ_w), with λ_b ← λ̂_b and c ← ĉ
        if λ_w^temp = λ̂_w then
            converged ← converged + 1
        else
            converged ← 0
        end if
        λ̂_w ← λ_w^temp
        select_λ_w ← FALSE
        select_λ_b ← TRUE
    else if select_λ_b then
        λ_b^temp ← StARS(λ_b), with λ_w ← λ̂_w and c ← ĉ
        if λ_b^temp = λ̂_b then
            converged ← converged + 1
        else
            converged ← 0
        end if
        λ̂_b ← λ_b^temp
        select_λ_b ← FALSE
    else
        c^temp ← StARS(c), with λ_w ← λ̂_w and λ_b ← λ̂_b
        if c^temp = ĉ then
            converged ← converged + 1
        else
            converged ← 0
        end if
        ĉ ← c^temp
        select_λ_w ← TRUE
        select_λ_b ← FALSE
    end if
    iter ← iter + 1
end while
Return (λ̂_w, λ̂_b, ĉ)
```

2. Dimensionality of the partitions of $\boldsymbol{W}_{\backslash i \backslash i}$

It is relevant to notice that the dimensions of the partitions of $\boldsymbol{W}_{\backslash i \backslash i}$ described in the manuscript depend on the index i. For example, we split $\boldsymbol{W}_{\backslash i \backslash i}$, of dimension $(p-1)*(p-1)$, into two rectangular vertical matrixes: $(\boldsymbol{W}_{\backslash i \backslash i})_X$ and $(\boldsymbol{W}_{\backslash i \backslash i})_Z$. Each one of these two vertical sections can have two different dimensions, depending on the position of the index i. The vertical section $(\boldsymbol{W}_{\backslash i \backslash i})_X$ has dimensions $(p-1)*p_x$ when the i-th row and column of $\boldsymbol{W}$ the algorithm is updating corresponds to a variable that belongs to the omics layer $\boldsymbol{Z}$. Otherwise, this first vertical section has dimensions $(p-1)*(p_x-1)$ if the i-th variable belongs to the to the omics layer $\boldsymbol{X}$. Comparably, $(\boldsymbol{W}_{\backslash i \backslash i})_Z$ can either have dimensions $(p-1)*(p_z-1)$ if the i-th variable belongs to omics layer $\boldsymbol{Z}$ and $(p-1)*p_z$ if it does not. Analogous reasoning can be applied for the dimension of the two partitions of the coefficient vector $\boldsymbol{\beta_i}$. $(\boldsymbol{\beta_i})_X$ can have either p_x or p_x-1 elements, while $(\boldsymbol{\beta_i})_Z$ can have either p_z or p_z-1 elements, depending on where the index i is located. This partitioning ensures that the collaborative penalty term in Equation 2 of the main manuscript remains well-defined across all omics layers, aligning dimensional consistency between the estimated coefficient vectors from omics layers $\boldsymbol{X}$ and $\boldsymbol{Z}$.

3. Scalability study of coglasso

To test for the scalability of our method, we measured the running time of *coglasso* in three settings of higher dimension: $p_X + p_Z = 1000$ (scenario S1), $p_X + p_Z = 1500$ (scenario S2), and $p_X + p_Z = 2000$ (scenario S3). Like for the second scenario presented in the Simulations section of the main text ($p_X + p_Z = 100$), the dimensions of X and Z are in a $4:1$ ratio, so Z is not fixed to 20 nodes anymore, but 200 (scenario S1), 300 (scenario S2), and 400 (scenario S3), while X has, respectively, 800, 1200, and 1600 nodes. In order to generate data of higher dimensions, some parameters of the original simulations had to be tweaked. In particular, the we modified the probabilities of intra-cluster connections both for X and Z, and the number of connections not to make the clusters completely disjoint. In scenario S1, the largest layer ($p_X = 800$) had a probability of *intra*-cluster connections of $\frac{1}{86}$ and 80 random connections to join the clusters, while the smallest layer ($p_Z = 200$) had a probability of $\frac{1}{31}$ and 35 random *inter*-cluster connections. In scenario S2, instead, the largest layer ($p_X = 1200$) had a probability of *intra*-cluster connections of $\frac{1}{131}$ and 100 random connections to join the clusters, while the smallest layer ($p_Z = 300$) had a probability of $\frac{1}{48}$ and 40 random *inter*-cluster connections. Finally, in scenario S3, the largest layer ($p_X = 1600$) had a probability of *intra*-cluster connections of $\frac{1}{175}$ and 150 random connections to join the clusters, while the smallest layer ($p_Z = 400$) had a probability of $\frac{1}{64}$ and 50 random *inter*-cluster connections. After this, the only parameters that changed were the small amounts added to the diagonal of the assembled Θ matrixes to guarantee positive semi-definitiveness. They were, respectively for the three scenarios S1 to S3, $\epsilon = 0.7$, $\epsilon = 0.7$, and $\epsilon = 0.6$. All other parameters described in the Simulations section stayed the same. Once the simulated data were available, we performed a runtime analysis of *coglasso* for $R = 10$ generated datasets for each scenario. In particular, as the computational performance of our method chiefly depends on the density of the estimated network, we explored two extreme settings: one for which the estimated networks were dense, and one

for which the estimated networks were very sparse. This allows to provide an impression of the boundary performances of *coglasso*, providing one of the slowest and fastest scenarios. To induce a dense network estimation, we chose low λ_w and λ_b values, namely: 10% of the highest absolute Pearson correlation value among the *within* correlations (λ_w), and among the *between* correlations (λ_b), measured separately for each input dataset. Moreover, as also Supplementary Figure S10 shows, c can also affect sparsity. As a consequence, to contribute to a higher network density, we set $c = 100$. To induce a sparse, yet not empty, network estimation, we chose high λ_w and λ_b values. In this case, we first generate a sequence of 10 λ_w and 10 λ_b values, analogously to how we generate them in the Simulations section of the main text, and we take the fourth highest (fourth most penalizing) values for both. Analogously to the dense scenario, we picked $c = 0.01$ to select a c value that could support the sparsity of the estimated networks without switching off collaboration. Notably, our strategy does not fix λ_w and λ_b to hand-picked values on purpose, as for each of the ten replicates analysed for each scenario the data are randomly generated, and a single penalty value could result in very different final densities for different datasets. Hence, our choice of adaptive values for our penalty parameters. Supplementary figure S6 shows the results of our scalability study of the three scenarios, reporting running times (top panels) and estimated network densities (bottom panels) for 10 replicates of each of the three scenarios, for both the dense setting (left panels) and the sparse setting (right panels). To compensate for fluctuations in machine performance, we performed three technical replicates of each of the ten replicates for each scenario, and reported the average runtime of the three in the plot. As the figure shows, large dense networks of 2000 nodes can take as long as 11 thousands seconds, which amounts to $\sim$ 3 hours. As such, when dealing with very large networks, we advise to avoid exploring regions of the hyperparameter space that lead to very dense networks, which, apart from the computational burden, make the biological interpretation of the estimated networks complex and potentially increase the number of false positive edges. Otherwise, we advise to pre-select a number of omics variables below 1500 to alleviate the computational burden. Finally, we highlight that the *coglasso* algorithm allows for parallelization of the exploration of the hyperparameter space, which is of our future interest. Regarding the specifications of the machine used for the runtime measure test, we used *R* version 4.4.2, on a Windows machine with 51.3 GB of RAM and processor Intel (R) Xeon (R) W-2235.

4. Comparison of the application of coglasso and glasso to the multi-omics study of sleep deprivation in mouse

In the main manuscript we use *coglasso* to reconstruct a multi-omics network from a study of sleep deprivation in mouse [Diessler et al., 2018]. For comparison, we reconstructed also a network with *glasso* [Friedman et al., 2008] using the same data, selecting the optimal λ value with *StARS* [Liu et al., 2010] among a pool of 30 values in the range $[0, 1]$. In particular, we used the implementation of the R package *huge*. *StARS* selected $\hat{\lambda} = 0.47$. Supplementary Figure S9 shows the selected *glasso* network. In the figure, it appears that *glasso* estimated a network with a lower density between omics layers, compared to the one estimated by *coglasso*. In particular, of all the estimated connections in the selected networks, the between connections amounted to 65% for *coglasso*, and to 8% for *glasso*. This resulted in the loss of important between connections estimated by *coglasso* that were previously validated in literature, including the connections: *Fos*-leucine; *Junb*-methionine; and *Junb*-histidine. This happened despite *glasso*'s network having more connections (1645 total connections, compared to the 1308 of *coglasso*'s network). Also the overlaps are limited, with the two networks sharing only 596 connections.

Table 1. Partial correlation values of *Cirbp*'s connections to its neighbourhood

Feature	Partial correlation	Quartile
Hsph1[1]	-0.081	Q4
P4ha1	-0.072	Q4
PC aa C36:3	-0.071	Q4
Pdia6[1]	-0.067	Q4
Manf[1]	-0.053	Q4
Stip1[1]	-0.047	Q3
Trp	-0.039	Q3
Dnajb11[1]	-0.029	Q3
Putrescine	-0.028	Q3
Spred1	-0.027	Q2
BC004004	0.027	Q2
Phe	-0.025	Q2
Creld2[1]	-0.019	Q2
Hspa5 [1,2]	-0.017	Q2
Spry4	-0.017	Q2
Hsp90b1[1,2]	-0.011	Q1
Pdia3[1]	-0.011	Q1
Dnajc3[1]	-0.011	Q1
Gfod1	-0.001	Q1
PC ae C32:2	-0.001	Q1

As for Figure 3 in the main text, partial correlations are grouped in quartiles of intensity, with Q4 being the quartile of the strongest connections. Features in italics are genes, while the others are metabolites.

[1]Involved in unfolded protein response (UPR) or protein folding.

[2]Its regulation by *Cirbp* is scientifically proven.

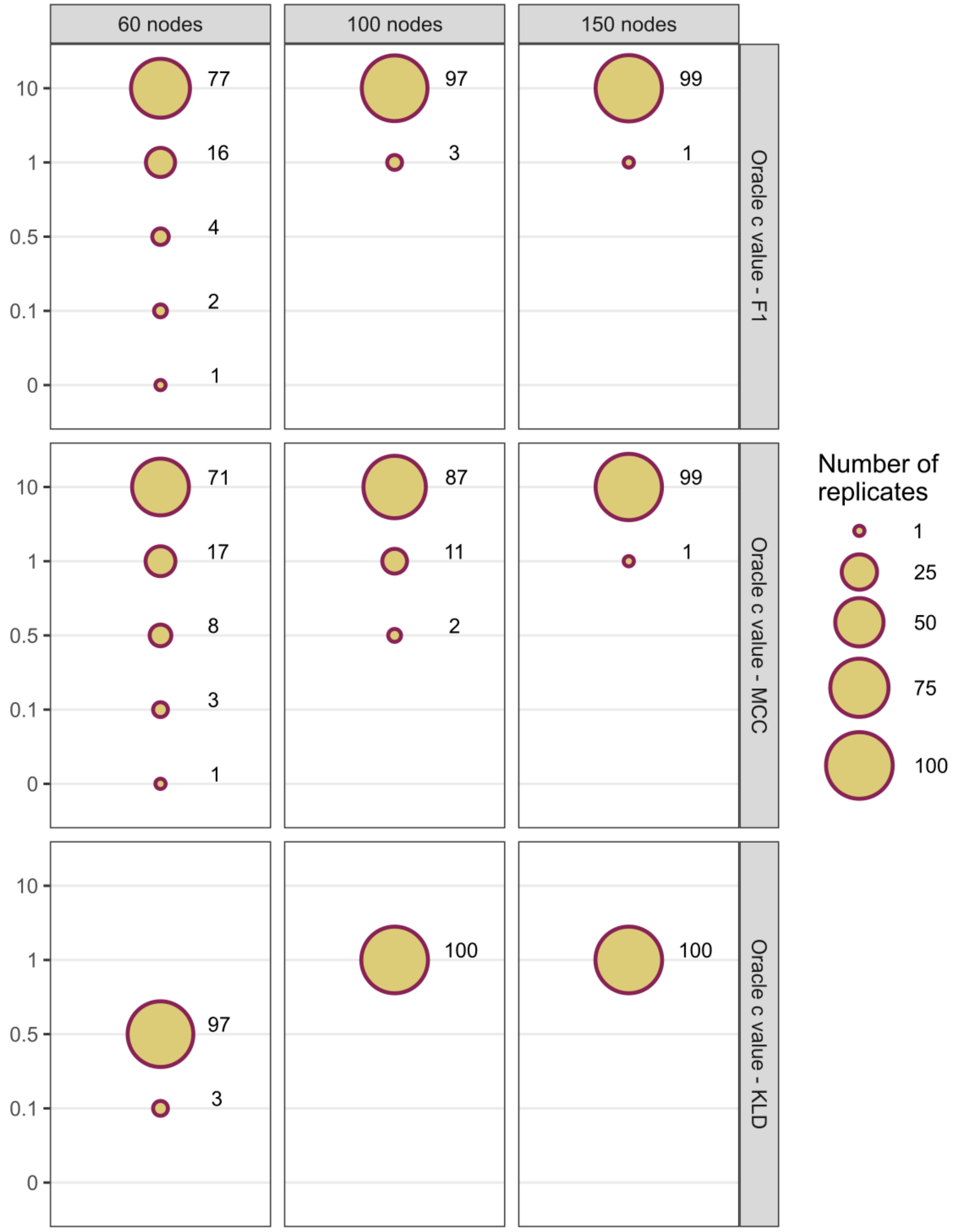

Supplementary Figure S2: Bubble plot of the c value of oracle *coglasso* networks in our simulated scenarios and according to the three measures used. The simulation scenarios are distributed along the columns, and the measures are distributed along rows. The size of the bubbles represents the proportion of the 100 replicates of each simulation scenario for which the c value indicated on the y-axis led to the best achieving *coglasso* network according to the corresponding performance measure. For each bubble, we report the associated number of replicates beside it. The c value is zero only for a single replicate and only according to F_1 and MCC in the least complex scenario. In the 99 remaining replicates of the least complex scenario for F_1 and MCC, and in all replicates for KLD, the c value of the best network is never zero. In both the more complex scenarios, the c of the best network is also never zero. This implies a role of collaboration in achieving the best network.

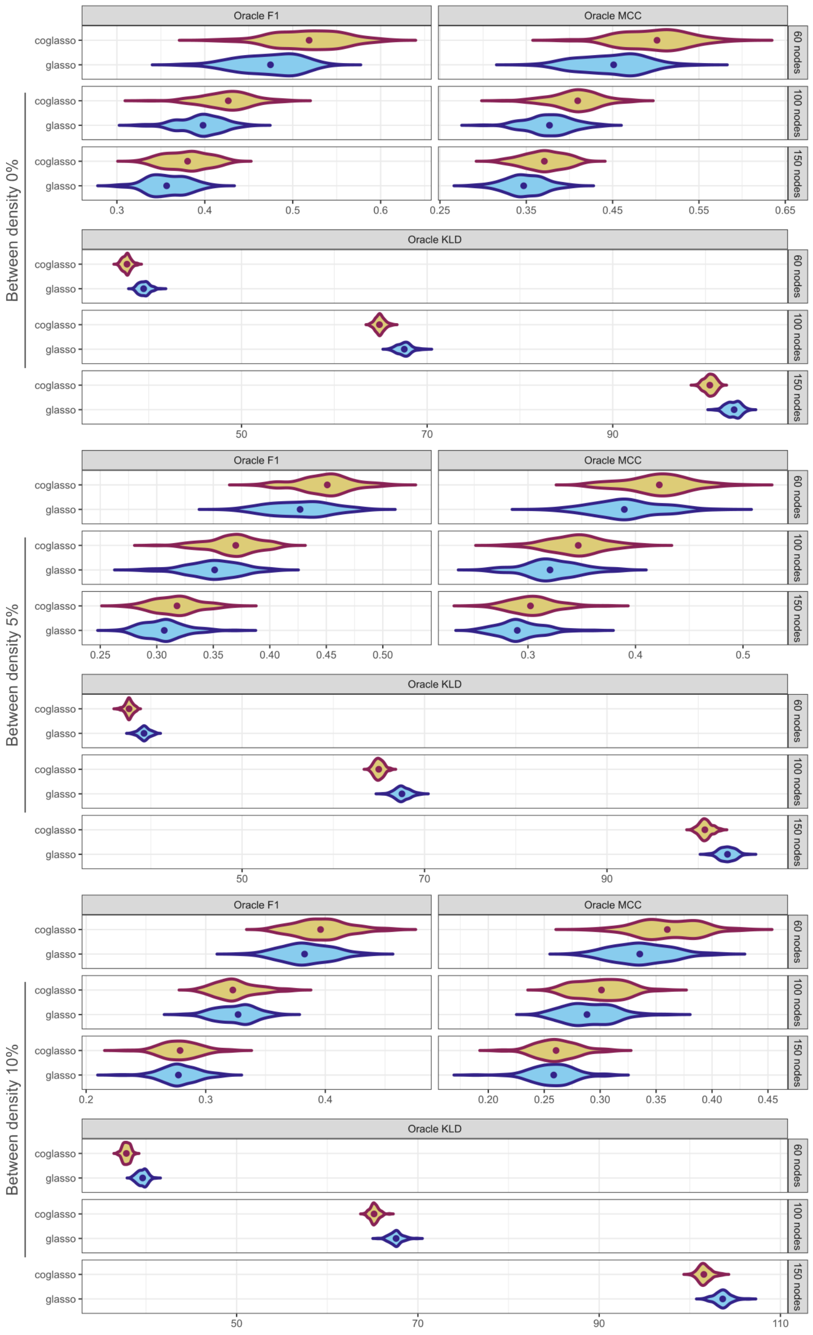

Supplementary Figure S3: Simulations run with different network densities between the simulated layers, specifically 0%, 5%, and 10%. In most scenarios, and according to all measures, *coglasso* shows advantage over *glasso*. As this scenarios encapsulate multiple levels of network complexity, in an attempt to emulate biological complexity, the two layers are of different dimensions (the smallest layer has always 20 nodes, while the largest layers have 40, 80, and 130 nodes, respectively, in the three scenarios). This may cause the largest layer to dominate the network estimation, exerting a strong bias. Even for the scenario with completely disconnected simulated layers, *coglasso* shows an advantage. Our hypothesis is that, even in sparser *between* scenarios, collaboration can still mitigate the bias induced in the network estimation by the presence of a larger layer. This is further shown in Supplementary Figure S4, where often the oracle c value is not 0.

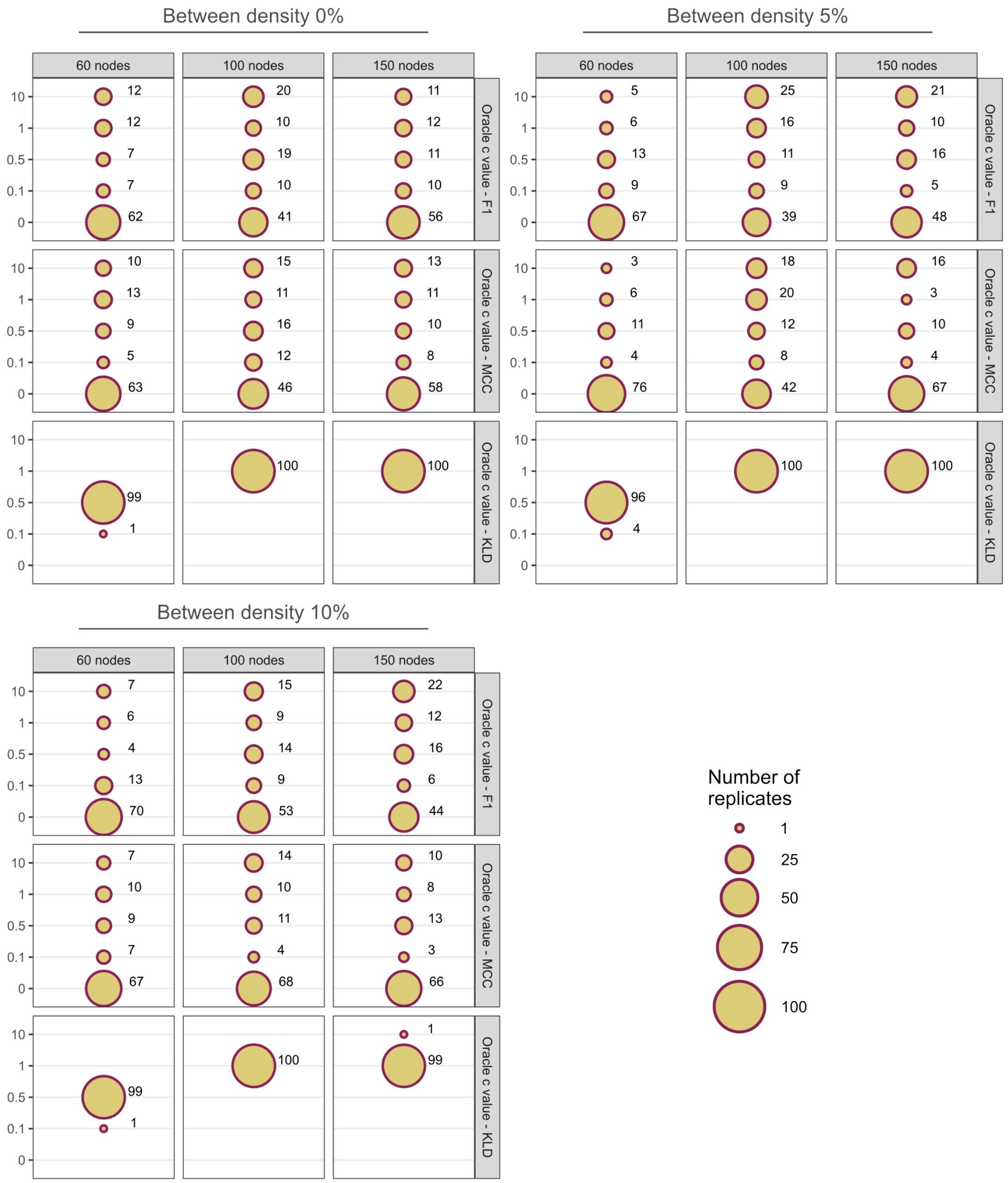

Supplementary Figure S4: Oracle c from simulations run with different between densities. The presence of oracle c values higher than 0 suggests that even with sparser between layer scenarios, collaboration can exert a positive role.

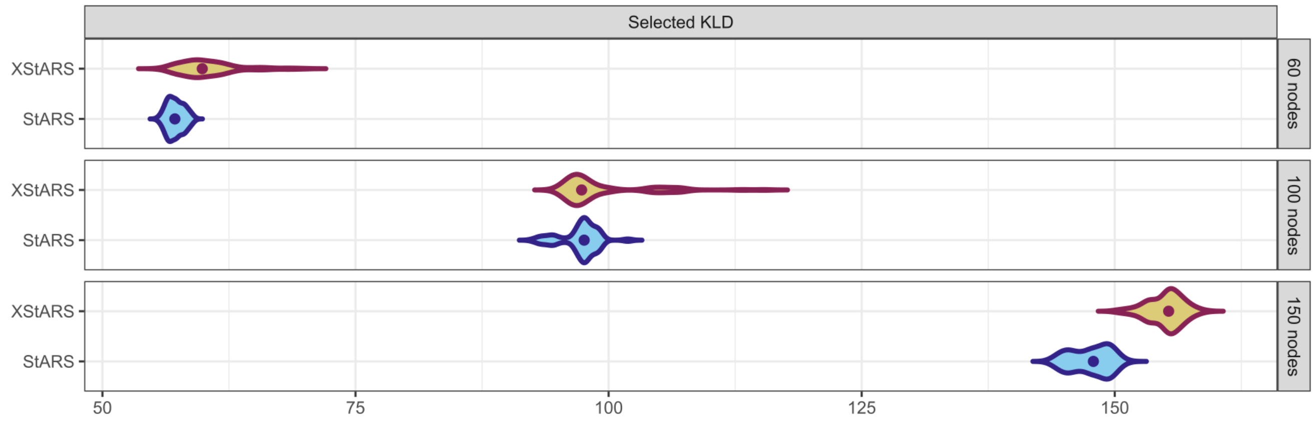

Supplementary Figure S5: Performance of model selection with *XStARS* for *coglasso* (in yellow-red) and *StARS* for *glasso* (in cyan-blue) over three increasingly complex and high-dimensional scenarios (networks with 60, 100, and 150 nodes) in terms of Kullback-Leibler divergence (KLD). The scenarios are distributed along the rows of the figure. The violins of the figure are composed of 100 data points, one for each replicate of the simulations, and the networks were reconstructed from datasets of $n = 50$ observations.

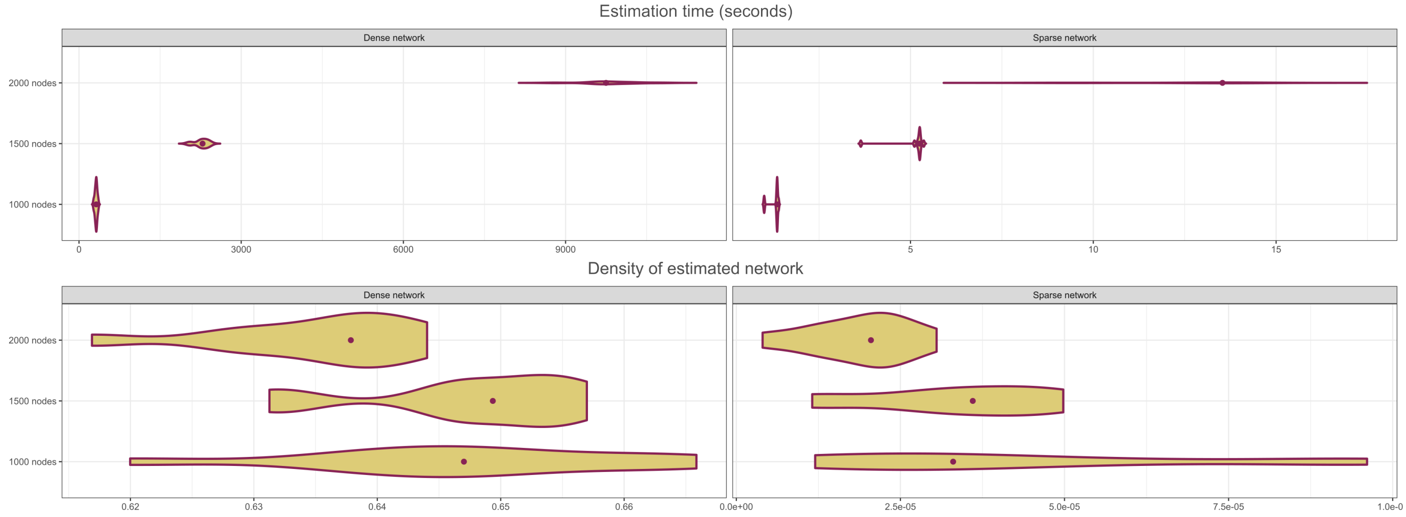

Supplementary Figure S6: Computational performance of *coglasso* for higher-dimensional network estimation (higher panels) compared to network density (lower panels), for dense networks (left panels) and sparse networks (right panels). In particular, we timed the estimation of *coglasso* $R = 10$ networks of 1000 nodes ($p_X = 800$, $p_Z = 200$), 1500 nodes ($p_X = 1200$, $p_Z = 300$), and 2000 nodes ($p_X = 1600$, $p_Z = 400$) in two scenarios of different densities. For the dense networks scenario (left panels) the density of the estimated network was set in the range $[62\% - 67\%]$ (lower-left panel) by choosing low λ_w and λ_b values, and setting $c = 100$, while for the sparse scenario (right panels) the density was kept to near zero by selecting high λ_w and λ_b values, and setting $c = 0.01$. These two scenarios give an impression of the slowest (left) and fastest (right) time performance of *coglasso*.

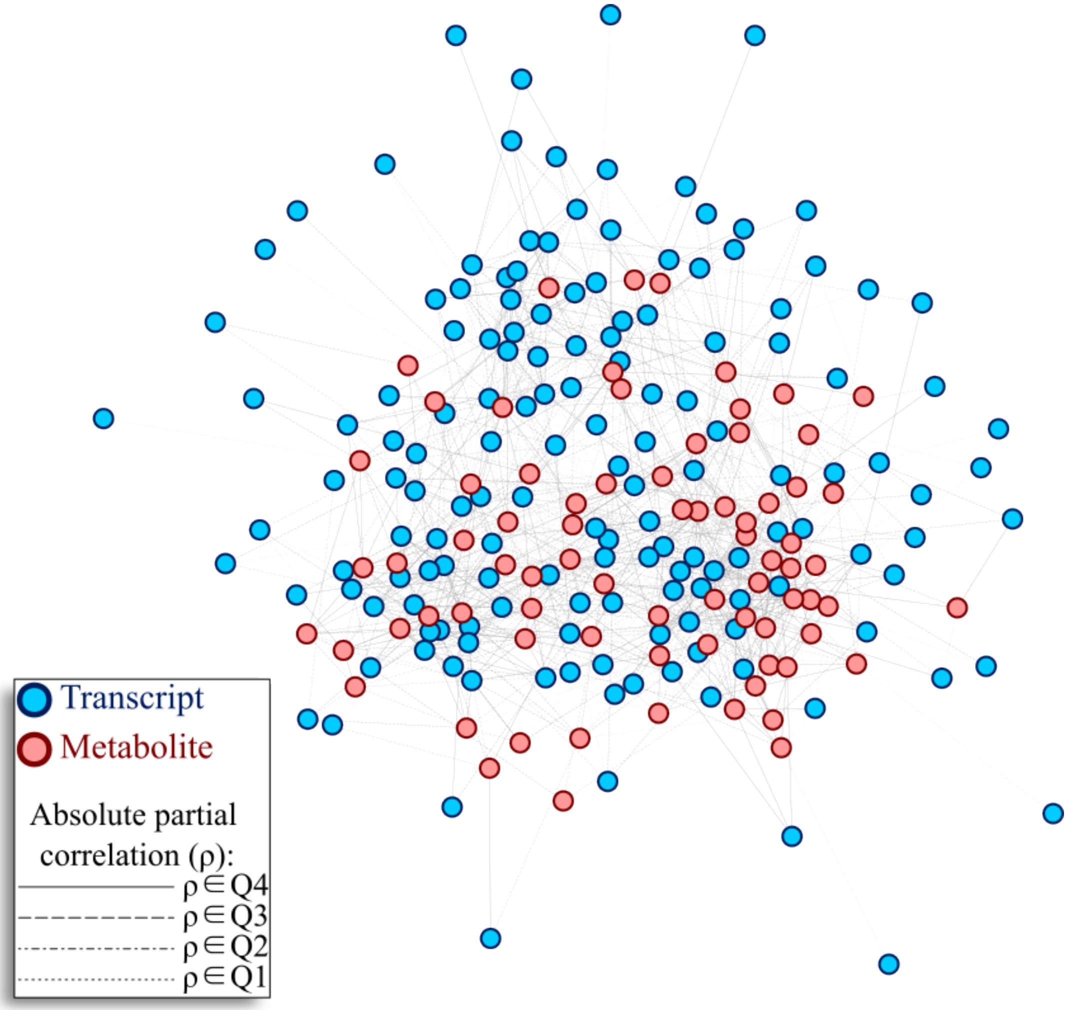

Supplementary Figure S7: Multi-omics network inferred using *coglasso*, selected by *XStARS* model selection. Blue nodes represent transcripts, while pink nodes represent metabolites. The fragmentation of an edge represents the absolute partial correlation related to the edge, according to the quartile they belong to. The strongest partial correlations are part of the fourth quartile, and are represented as continuous lines. The lower quartiles are progressively represented with more fragmented lines, until the fully dotted line of the first quartile. The figure shows how the two omics layers are highly blended, representing a large amount of connections shared among them.

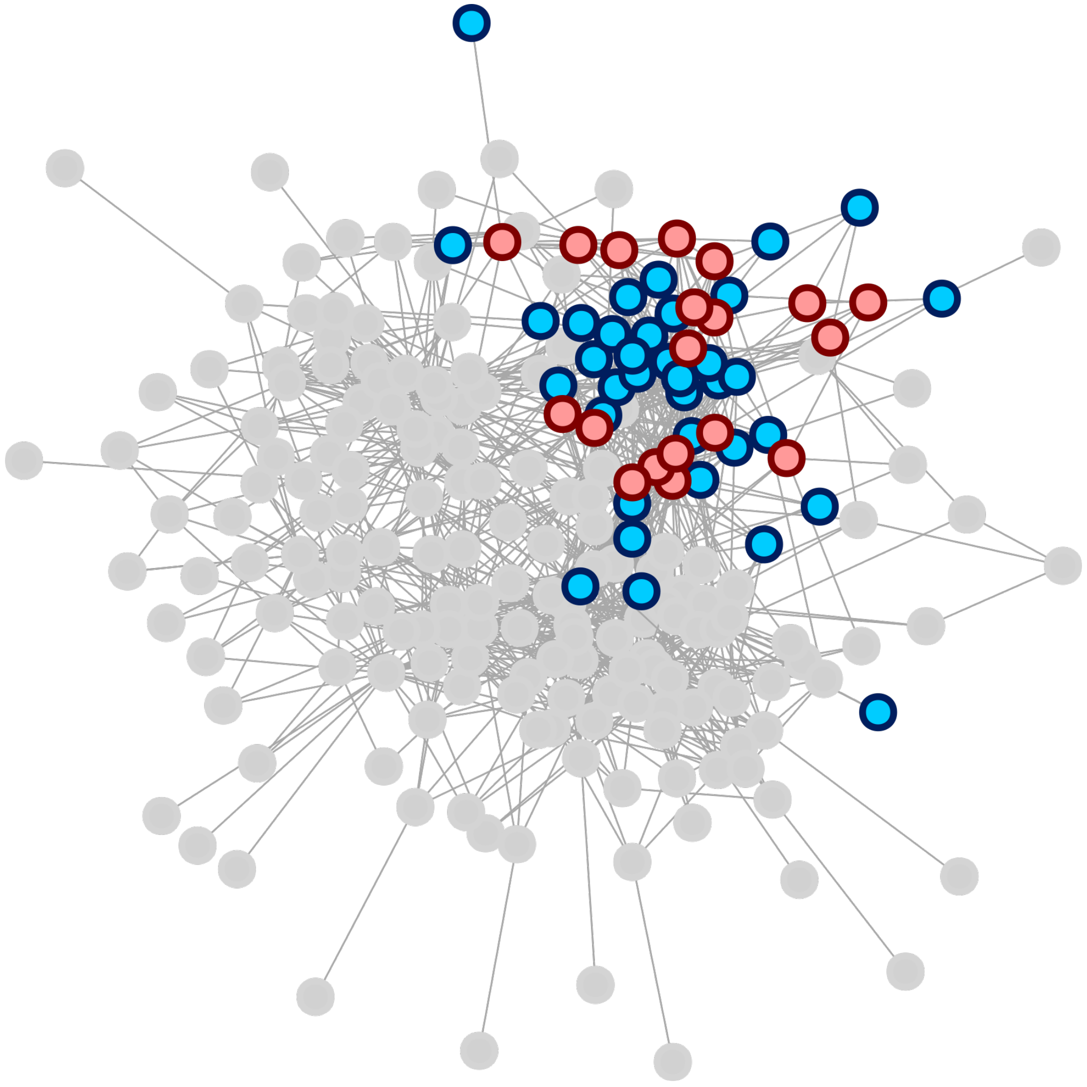

Supplementary Figure S8: Multi-omics network inferred using *coglasso*. The nodes belonging to the second largest node community are highlighted here.

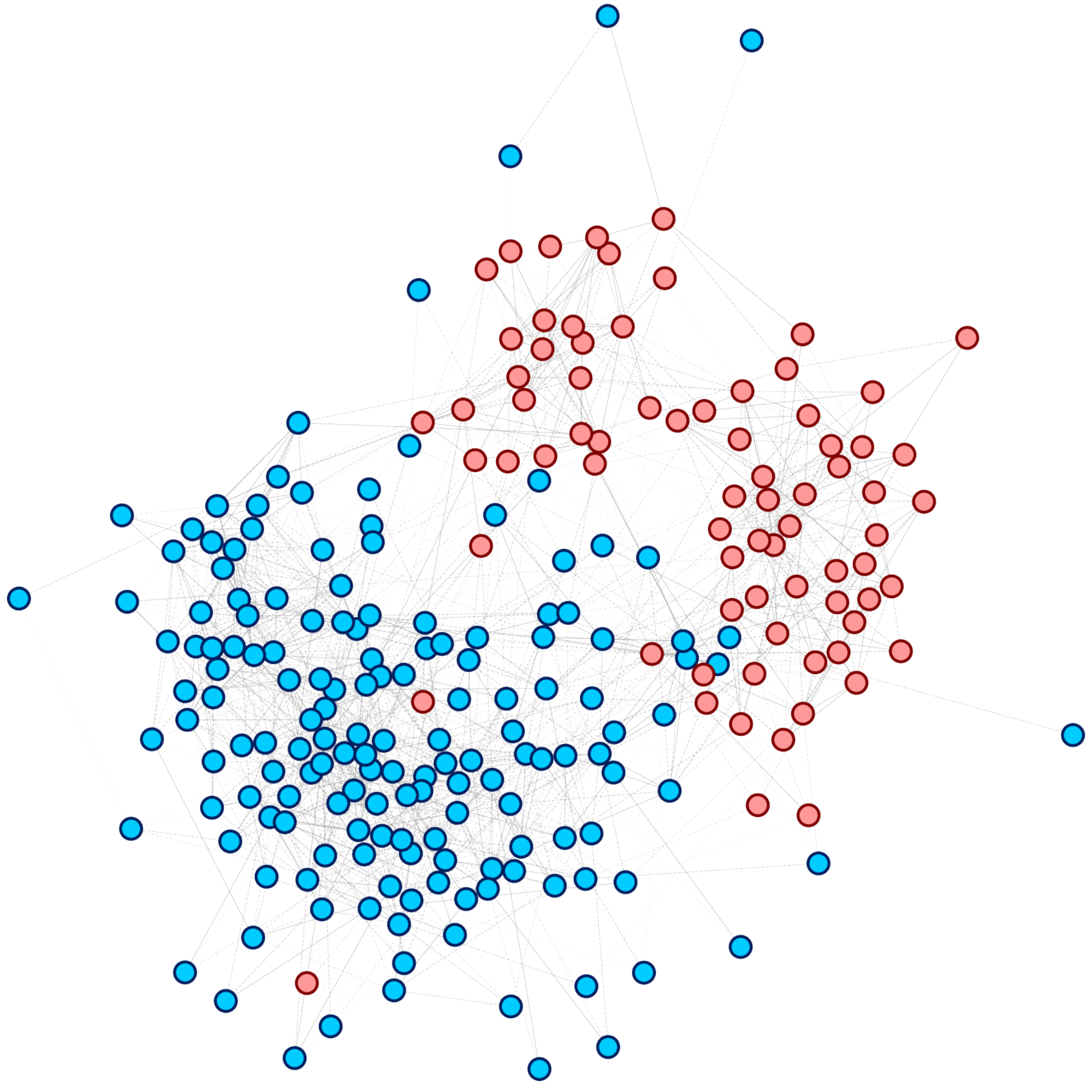

Supplementary Figure S9: The *glasso*-estimated network. It is evident that the density between the transcriptomic and the metabolomic layer is lower for this network than for the *coglasso*-estimated network.

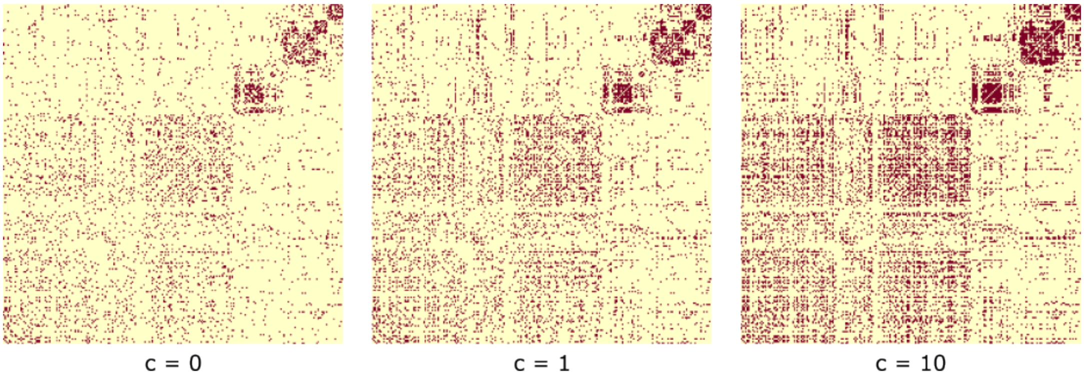

Supplementary Figure S10: The structure of a *coglasso*-estimated network for different c values (0, 1, and 10), keeping $\lambda_w = 0.39$ and $\lambda_b = 0.36$. For each c value, an image of the adjacency matrix is provided. A red dot in entry (i, j) of the matrix represents a connection between variables i and j. In this example, increasing c leads to a denser estimated network.

Appendix A: Derivation of coglasso coordinate descent update rule

In this appendix, we report the original *coglasso* objective function, we develop it and we minimize it with respect to the jth component of the vector $\boldsymbol{\beta_i}$. Here is the objective function:

$$F_{\boldsymbol{\Lambda},\boldsymbol{W}_{\setminus i\setminus i}}(\boldsymbol{\beta_i}) = \frac{1}{2}\left\|(\boldsymbol{W}_{\setminus i\setminus i})^{-1/2}\boldsymbol{S}_i - (\boldsymbol{W}_{\setminus i\setminus i})^{1/2}\boldsymbol{\beta_i}\right\|^2 + \frac{c}{2}\left\|(\boldsymbol{W}_{\setminus i\setminus i})^{1/2}_X(\boldsymbol{\beta_i})_X - (\boldsymbol{W}_{\setminus i\setminus i})^{1/2}_Z(\boldsymbol{\beta_i})_Z\right\|^2 + \|\boldsymbol{\Lambda}_i \odot \boldsymbol{\beta}_i\|_1$$

For an explanation of the elements of the objective function, we refer the reader to the Materials and Methods section of the manuscript. Now, developing the elements of the objective function:

$$\begin{aligned}F_{\boldsymbol{\Lambda},\boldsymbol{W}_{\setminus i\setminus i}}(\boldsymbol{\beta_i}) =& \frac{1}{2}\boldsymbol{S}_i^T(\boldsymbol{W}^{-1/2}_{\setminus i\setminus i})^T\boldsymbol{W}^{-1/2}_{\setminus i\setminus i}\boldsymbol{S}_i \\ &+ \frac{1}{2}\boldsymbol{\beta_i}^T(\boldsymbol{W}^{1/2}_{\setminus i\setminus i})^T\boldsymbol{W}^{1/2}_{\setminus i\setminus i}\boldsymbol{\beta_i} \\ &- \boldsymbol{\beta_i}^T(\boldsymbol{W}^{-1/2}_{\setminus i\setminus i})^T\boldsymbol{W}^{1/2}_{\setminus i\setminus i}\boldsymbol{S}_i \\ &+ \frac{c}{2}(\boldsymbol{\beta_i})_X^T(\boldsymbol{W}^{1/2}_{\setminus i\setminus i})_X^T(\boldsymbol{W}^{1/2}_{\setminus i\setminus i})_X(\boldsymbol{\beta_i})_X \\ &+ \frac{c}{2}(\boldsymbol{\beta_i})_Z^T(\boldsymbol{W}^{1/2}_{\setminus i\setminus i})_Z^T(\boldsymbol{W}^{1/2}_{\setminus i\setminus i})_Z(\boldsymbol{\beta_i})_Z \\ &- \frac{c}{2}(\boldsymbol{\beta_i})_X^T(\boldsymbol{W}^{1/2}_{\setminus i\setminus i})_X^T(\boldsymbol{W}^{1/2}_{\setminus i\setminus i})_Z(\boldsymbol{\beta_i})_Z \\ &- \frac{c}{2}(\boldsymbol{\beta_i})_Z^T(\boldsymbol{W}^{1/2}_{\setminus i\setminus i})_Z^T(\boldsymbol{W}^{1/2}_{\setminus i\setminus i})_X(\boldsymbol{\beta_i})_X \\ &+ \|\boldsymbol{\Lambda}_i \odot \boldsymbol{\beta}_i\|_1\end{aligned}$$

Considering that the matrix $\boldsymbol{W}_{\setminus i\setminus i}$ can be divided in the following block matrix:

$$\boldsymbol{W}_{\setminus i\setminus i} = \begin{pmatrix}(\boldsymbol{W}_{\setminus i\setminus i})_{XX} & (\boldsymbol{W}_{\setminus i\setminus i})_{XZ} \\ (\boldsymbol{W}_{\setminus i\setminus i})^T_{XZ} & (\boldsymbol{W}_{\setminus i\setminus i})_{ZZ}\end{pmatrix}$$

and taking into account the equivalences shown in Appendix B, it's easy to see what follows:

- $(\boldsymbol{W}^{-1/2}_{\setminus i\setminus i})^T\boldsymbol{W}^{-1/2}_{\setminus i\setminus i} = \Theta_{\setminus i\setminus i}$
- $(\boldsymbol{W}^{1/2}_{\setminus i\setminus i})^T_X(\boldsymbol{W}^{1/2}_{\setminus i\setminus i})_X = (\boldsymbol{W}_{\setminus i\setminus i})_{XX}$ and $(\boldsymbol{W}^{1/2}_{\setminus i\setminus i})^T_Z(\boldsymbol{W}^{1/2}_{\setminus i\setminus i})_Z = (\boldsymbol{W}_{\setminus i\setminus i})_{ZZ}$
- $(\boldsymbol{W}^{1/2}_{\setminus i\setminus i})^T_X(\boldsymbol{W}^{1/2}_{\setminus i\setminus i})_Z = (\boldsymbol{W}_{\setminus i\setminus i})_{XZ}$ and $(\boldsymbol{W}^{1/2}_{\setminus i\setminus i})^T_Z(\boldsymbol{W}^{1/2}_{\setminus i\setminus i})_X = (\boldsymbol{W}_{\setminus i\setminus i})^T_{XZ}$

Considering the previous rewritings, the objective function can be simplified to the following form:

$$\begin{aligned}F_{\boldsymbol{\Lambda},\boldsymbol{W}_{\setminus i\setminus i}}(\boldsymbol{\beta_i}) =& \frac{1}{2}\boldsymbol{S}_i^T\boldsymbol{\Theta}_{\setminus i\setminus i}\boldsymbol{S}_i + \frac{1}{2}\boldsymbol{\beta_i}^T\boldsymbol{W}_{\setminus i\setminus i}\boldsymbol{\beta_i} - \boldsymbol{\beta_i}^T\boldsymbol{S}_i \\ &+ \frac{c}{2}(\boldsymbol{\beta_i})_X^T(\boldsymbol{W}_{\setminus i\setminus i})_{XX}(\boldsymbol{\beta_i})_X \\ &+ \frac{c}{2}(\boldsymbol{\beta_i})_Z^T(\boldsymbol{W}_{\setminus i\setminus i})_{ZZ}(\boldsymbol{\beta_i})_Z \\ &- \frac{c}{2}(\boldsymbol{\beta_i})_X^T(\boldsymbol{W}_{\setminus i\setminus i})_{XZ}(\boldsymbol{\beta_i})_Z \\ &- \frac{c}{2}(\boldsymbol{\beta_i})_Z^T(\boldsymbol{W}_{\setminus i\setminus i})^T_{XZ}(\boldsymbol{\beta_i})_X + \|\boldsymbol{\Lambda}_i \odot \boldsymbol{\beta}_i\|_1\end{aligned}$$

We now define the matrix of off-diagonal blocks obtained from $\boldsymbol{W}_{\setminus i\setminus i}$ as:

$$\boldsymbol{W}^{ODB}_{\setminus i\setminus i} = \begin{pmatrix}\boldsymbol{0}_{XX} & (\boldsymbol{W}_{\setminus i\setminus i})_{XZ} \\ (\boldsymbol{W}_{\setminus i\setminus i})^T_{XZ} & \boldsymbol{0}_{ZZ}\end{pmatrix}$$

It's now straightforward to see that

$$(\boldsymbol{\beta_i})_X^T(\boldsymbol{W}_{\setminus i\setminus i})_{XZ}(\boldsymbol{\beta_i})_Z + (\boldsymbol{\beta_i})_Z^T(\boldsymbol{W}_{\setminus i\setminus i})^T_{XZ}(\boldsymbol{\beta_i})_X = \boldsymbol{\beta_i}^T\boldsymbol{W}^{ODB}_{\setminus i\setminus i}\boldsymbol{\beta_i},$$

and considering that

$$\begin{aligned}&(\boldsymbol{\beta_i})_X^T(\boldsymbol{W}_{\setminus i\setminus i})_{XX}(\boldsymbol{\beta_i})_X + (\boldsymbol{\beta_i})_Z^T(\boldsymbol{W}_{\setminus i\setminus i})_{ZZ}(\boldsymbol{\beta_i})_Z \\ &= \boldsymbol{\beta_i}^T\boldsymbol{W}_{\setminus i\setminus i}\boldsymbol{\beta_i} - (\boldsymbol{\beta_i})_X^T(\boldsymbol{W}_{\setminus i\setminus i})_{XZ}(\boldsymbol{\beta_i})_Z - (\boldsymbol{\beta_i})_Z^T(\boldsymbol{W}_{\setminus i\setminus i})^T_{XZ}(\boldsymbol{\beta_i})_X \\ &= \boldsymbol{\beta_i}^T\boldsymbol{W}_{\setminus i\setminus i}\boldsymbol{\beta_i} - \boldsymbol{\beta_i}^T\boldsymbol{W}^{ODB}_{\setminus i\setminus i}\boldsymbol{\beta_i},\end{aligned}$$

the objective function becomes:

$$\begin{aligned}F_{\boldsymbol{\Lambda},\boldsymbol{W}_{\setminus i\setminus i}}(\boldsymbol{\beta_i}) =& \frac{1}{2}\boldsymbol{S}_i^T\boldsymbol{\Theta}_{\setminus i\setminus i}\boldsymbol{S}_i + \frac{1+c}{2}\boldsymbol{\beta_i}^T\boldsymbol{W}_{\setminus i\setminus i}\boldsymbol{\beta_i} \\ &- c\boldsymbol{\beta_i}^T\boldsymbol{W}^{ODB}_{\setminus i\setminus i}\boldsymbol{\beta_i} - \boldsymbol{\beta_i}^T\boldsymbol{S}_i + \|\boldsymbol{\Lambda}_i \odot \boldsymbol{\beta}_i\|_1\end{aligned}$$

Now we minimize with respect to the jth element of $\boldsymbol{\beta_i}$, symbolized here by β_j. Of the dot products in the previous equation, we display in the next only the terms dependent on β_j. Moreover, we remark that all the penalty coefficients that are part of $\boldsymbol{\Lambda}$ are defined to be positive.

$$\begin{aligned}\frac{\partial F_{\boldsymbol{\Lambda},\boldsymbol{W}_{\setminus i\setminus i}}(\boldsymbol{\beta_i})}{\partial\beta_j} =& \frac{\partial}{\partial\beta_j}\Bigg\{\frac{1+c}{2}\left(\beta_j^2(\boldsymbol{W}_{\setminus i\setminus i})_{jj} + 2\beta_j\sum_{k\neq j}(\boldsymbol{W}_{\setminus i\setminus i})_{jk}\beta_k\right) \\ &- c\left(\beta_j^2(\boldsymbol{W}_{\setminus i\setminus i})^{ODB}_{jj} + 2\beta_j\sum_{k\neq j}(\boldsymbol{W}_{\setminus i\setminus i})^{ODB}_{jk}\beta_k\right) \\ &- \beta_j S_{ji} + \|\boldsymbol{\Lambda}_i \odot \boldsymbol{\beta}_i\|_1\Bigg\} \\ =& (1+c)\beta_j(\boldsymbol{W}_{\setminus i\setminus i})_{jj} + (1+c)\sum_{k\neq j}(\boldsymbol{W}_{\setminus i\setminus i})_{jk}\beta_k \\ &- 2c\sum_{k\neq j}(\boldsymbol{W}_{\setminus i\setminus i})^{ODB}_{jk}\beta_k - S_{ji} + \Lambda_{ji}\frac{\partial|\beta_j|}{\partial\beta_j}\end{aligned}$$

Notice how the term containing $(\boldsymbol{W}_{\setminus i\setminus i})^{ODB}_{jj}$ disappears, as all diagonal elements of $\boldsymbol{W}^{ODB}_{\setminus i\setminus i}$ are zero. In particular, with j being part of the generic dataset A, that could either be X or Z:

$$\begin{aligned}(1+c)\sum_{k\neq j}(\boldsymbol{W}_{\setminus i\setminus i})_{jk}\beta_k =& (1+c)\sum_{k\notin A}(\boldsymbol{W}_{\setminus i\setminus i})_{jk}\beta_k \\ &+ (1+c)\sum_{\substack{k\in A\\k\neq j}}(\boldsymbol{W}_{\setminus i\setminus i})_{jk}\beta_k \\ 2c\sum_{k\neq j}(\boldsymbol{W}_{\setminus i\setminus i})^{ODB}_{jk}\beta_k =& 2c\sum_{k\notin A}(\boldsymbol{W}_{\setminus i\setminus i})^{ODB}_{jk}\beta_k \\ =& 2c\sum_{k\notin A}(\boldsymbol{W}_{\setminus i\setminus i})_{jk}\beta_k\end{aligned}$$

Again, here all the elements $(\boldsymbol{W}_{\setminus i\setminus i})^{ODB}_{jk}$ are zero for every $k \in A$, since the diagonal blocks of the matrix are zero, and all the other elements coincide with the corresponding elements in

$\boldsymbol{W}_{\setminus i \setminus i}$. Hence, setting $\frac{\partial F_{\boldsymbol{\Lambda}, \boldsymbol{W}_{\setminus i \setminus i}}(\boldsymbol{\beta_i})}{\partial \beta_j} = 0$, we can rewrite the equation as:

$$\beta_j = \frac{1}{(1+c)(\boldsymbol{W}_{\setminus i \setminus i})_{jj}} \Bigg\{ S_{ji} - (1+c) \sum_{k \notin A} (\boldsymbol{W}_{\setminus i \setminus i})_{jk} \beta_k - (1+c) \sum_{\substack{k \in A \\ k \neq j}} (\boldsymbol{W}_{\setminus i \setminus i})_{jk} \beta_k + 2c \sum_{k \notin A} (\boldsymbol{W}_{\setminus i \setminus i})_{jk} \beta_k - \Lambda_{ji} \frac{\partial |\beta_j|}{\partial \beta_j} \Bigg\},$$

which, for $\alpha = \frac{1}{1+c}$ and simplifying, can be written as:

$$\beta_j = \frac{1}{(\boldsymbol{W}_{\setminus i \setminus i})_{jj}} \Bigg\{ \alpha S_{ji} - \sum_{\substack{k \in A \\ k \neq j}} (\boldsymbol{W}_{\setminus i \setminus i})_{jk} \beta_k - \alpha(1-c) \sum_{k \notin A} (\boldsymbol{W}_{\setminus i \setminus i})_{jk} \beta_k - \alpha \Lambda_{ji} \frac{\partial |\beta_j|}{\partial \beta_j} \Bigg\}.$$

From here, by applying the soft-threshold operator as seen in Friedman et al. [2007], one can straightforwardly obtain Algorithm 1 as shown in Section 3.

Appendix B: Equivalences involving the estimated variance-covariance matrix

It can be proven that the squared root of a symmetric positive semidefinite matrix is a symmetric positive semidefinite matrix itself. Nevertheless, such proof is out of the scope of this manuscript. Given this statement, since both $\boldsymbol{W}_{\setminus i \setminus i}$ and $\boldsymbol{\Theta}_{\setminus i \setminus i}$ are symmetric positive semidefinite, so are their squared roots. This allows us to prove the following two sets of equivalences:

$$\begin{pmatrix} (\boldsymbol{W}_{\setminus i \setminus i})_{XX} & (\boldsymbol{W}_{\setminus i \setminus i})_{XZ} \\ (\boldsymbol{W}_{\setminus i \setminus i})_{XZ}^T & (\boldsymbol{W}_{\setminus i \setminus i})_{ZZ} \end{pmatrix} = \boldsymbol{W}_{\setminus i \setminus i} = \boldsymbol{W}_{\setminus i \setminus i}^{1/2} \boldsymbol{W}_{\setminus i \setminus i}^{1/2}$$
$$= (\boldsymbol{W}_{\setminus i \setminus i}^{1/2})^T \boldsymbol{W}_{\setminus i \setminus i}^{1/2} = \begin{pmatrix} (\boldsymbol{W}_{\setminus i \setminus i}^{1/2})_X^T \\ (\boldsymbol{W}_{\setminus i \setminus i}^{1/2})_Z^T \end{pmatrix} \begin{pmatrix} (\boldsymbol{W}_{\setminus i \setminus i}^{1/2})_X & (\boldsymbol{W}_{\setminus i \setminus i}^{1/2})_Z \end{pmatrix}$$
$$= \begin{pmatrix} (\boldsymbol{W}_{\setminus i \setminus i}^{1/2})_X^T (\boldsymbol{W}_{\setminus i \setminus i}^{1/2})_X & (\boldsymbol{W}_{\setminus i \setminus i}^{1/2})_X^T (\boldsymbol{W}_{\setminus i \setminus i}^{1/2})_Z \\ (\boldsymbol{W}_{\setminus i \setminus i}^{1/2})_Z^T (\boldsymbol{W}_{\setminus i \setminus i}^{1/2})_X & (\boldsymbol{W}_{\setminus i \setminus i}^{1/2})_Z^T (\boldsymbol{W}_{\setminus i \setminus i}^{1/2})_Z \end{pmatrix} \quad (1)$$

$$\Theta_{\setminus i \setminus i} = \boldsymbol{W}_{\setminus i \setminus i}^{-1} = \boldsymbol{W}_{\setminus i \setminus i}^{-1/2} \boldsymbol{W}_{\setminus i \setminus i}^{-1/2} = (\boldsymbol{W}_{\setminus i \setminus i}^{-1/2})^T \boldsymbol{W}_{\setminus i \setminus i}^{-1/2} \quad (2)$$

References

Shanaz Diessler, Maxime Jan, Yann Emmenegger, Nicolas Guex, Benita Middleton, Debra J. Skene, Mark Ibberson, Frederic Burdet, Lou Götz, Marco Pagni, Martial Sankar, Robin Liechti, Charlotte N. Hor, Ioannis Xenarios, and Paul Franken. A systems genetics resource and analysis of sleep regulation in the mouse. *PLOS Biology*, 16(8):e2005750, August 2018. ISSN 1545-7885. doi: 10.1371/journal.pbio.2005750. Publisher: Public Library of Science.

Jerome Friedman, Trevor Hastie, Holger Höfling, and Robert Tibshirani. Pathwise coordinate optimization. *The Annals of Applied Statistics*, 1(2):302–332, December 2007. ISSN 1932-6157, 1941-7330. doi: 10.1214/07-AOAS131.

Jerome Friedman, Trevor Hastie, and Robert Tibshirani. Sparse inverse covariance estimation with the graphical lasso. *Biostatistics*, 9(3):432–441, July 2008. ISSN 1465-4644. doi: 10.1093/biostatistics/kxm045.

Han Liu, Kathryn Roeder, and Larry Wasserman. Stability Approach to Regularization Selection (StARS) for High Dimensional Graphical Models, June 2010. arXiv:1006.3316 [stat].
